# Haplotype block analysis of an Argentinean hexaploid wheat collection and GWAS for yield components and adaptation

**DOI:** 10.1186/s12870-019-2015-4

**Published:** 2019-12-16

**Authors:** Silvana Marisol Luján Basile, Ignacio Abel Ramírez, Juan Manuel Crescente, Maria Belén Conde, Melina Demichelis, Pablo Abbate, William John Rogers, Ana Clara Pontaroli, Marcelo Helguera, Leonardo Sebastián Vanzetti

**Affiliations:** 1 Laboratorio de Biología Funcional y Biotecnología (BIOLAB)-INBIOTEC-CONICET, Facultad de Agronomía, UNCPBA., Av. República de Italia, Azul, 7300 Argentina; 2 Unidad Integrada Balcarce Facultad de Ciencias Agrarias, Universidad Nacional de Mar del Plata - Estación Experimental Agropecuaria Balcarce, Instituto Nacional de Tecnología, Ruta 226, km 73.5, Balcarce, 24105 Argentina; 3 Laboratorio de Biotecnología, EEA INTA Marcos Juárez, Grupo Biotecnología y Recursos Genéticos, Instituto Nacional de Tecnología Agropecuaria, Ruta 12 s/n, Marcos Juárez, 2580 Argentina; 4 Consejo Nacional de Investigaciones Científicas y Técnicas (CONICET)., Buenos Aires, Argentina

**Keywords:** Wheat, Haplotype, GWAS, Heading date, Plant height, Thousand grain weight, Grain number per spike, Fruiting efficiency at harvest

## Abstract

**Background:**

Increasing wheat (*Triticum aestivum* L.) production is required to feed a growing human population. In order to accomplish this task a deeper understanding of the genetic structure of cultivated wheats and the detection of genomic regions significantly associated with the regulation of important agronomic traits are necessary steps. To better understand the genetic basis and relationships of adaptation and yield related traits, we used a collection of 102 Argentinean hexaploid wheat cultivars genotyped with the 35k SNPs array, grown from two to six years in three different locations. Based on SNPs data and gene-related molecular markers, we performed a haplotype block characterization of the germplasm and a genome-wide association study (GWAS).

**Results:**

The genetic structure of the collection revealed four subpopulations, reflecting the origin of the germplasm used by the main breeding programs in Argentina. The haplotype block characterization showed 1268 blocks of different sizes spread along the genome, including highly conserved regions like the 1BS chromosome arm where the 1BL/1RS wheat/rye translocation is located. Based on GWAS we identified ninety-seven chromosome regions associated with heading date, plant height, thousand grain weight, grain number per spike and fruiting efficiency at harvest (FEh). In particular FEh stands out as a promising trait to raise yield potential in Argentinean wheats; we detected fifteen haplotypes/markers associated with increased FEh values, eleven of which showed significant effects in all three evaluated locations. In the case of adaptation, the *Ppd-D1* gene is consolidated as the main determinant of the life cycle of Argentinean wheat cultivars.

**Conclusion:**

This work reveals the genetic structure of the Argentinean hexaploid wheat germplasm using a wide set of molecular markers anchored to the Ref Seq v1.0. Additionally GWAS detects chromosomal regions (haplotypes) associated with important yield and adaptation components that will allow improvement of these traits through marker-assisted selection.

## Background

World population is projected to grow to nearly 10 billion by 2050 and more than 11 billion by 2100 and, on a global scale, agriculture expansion has slowed down and production increases have been achieved mainly through agricultural intensification [1]. Satisfying the increasing feed and food demand will mainly come from yield improvement: it will be required that wheat yield (and other staple crops) be increased by at least 50% in the next few decades [2], which will depend, among other components, on improving yield potential [3]. This scenario becomes more complex if we consider that each degree-Celsius increase in global mean temperature would, on average, reduce global and in particular Argentinean wheat yields by 6.0% [4, 5]. Furthermore, crop management will need to be environmentally more sustainable in the future [1]. Under this restrictive context, yield genetic gain will be required to increase by 1.16–1.31% per year to satisfy the projected demand of cereals for food, feed and biofuels by 2050 [6]. Unfortunately, genetic gains reported in wheat from different countries for the last decades seem to have been increasing less than required [7–9].

In Argentina, with 4.46 Mha, wheat is the third most important crop in terms of planted area after soybean and corn, average 2012-2016 [10], spanning a wide range of environments. Since 1999 the genetic gain of local cultivars has shown signs of stagnation, as indicated by genetic gain values of only 0.18% per year, mostly explained by a stabilization in grain number per unit area without changes in grain weight. Modern cultivars have increased the number of grains per spike without changes in spike number per unit area; fruiting efficiency (a.k.a. spike fertility index) was the trait that better explained the changes in grain number per unit area, and its improvement might be a way to promote increments in grain number without penalization in grain weight [11–13].

Quantitative trait locus (QTL) mapping is currently key for understanding the genetic basis of complex traits [14], including yield components and adaptation. Cost-effective, high-throughput genotyping tools for genetic study, such as single nucleotide polymorphism (SNP) arrays, are now available for all major crop species, including wheat [15], enabling the characterisation of (i) genome-wide population diversity and structure; (ii) selective sweeps and directional selection; and (iii) marker-trait relationships in genome-wide association studies (GWAS) [16, 17]. Nonetheless, in this latter context, certain limitations are found: (i) SNPs only provide biallelic information [18]; and (ii) associations are likely to be due to loci in linkage disequilibrium (LD) with a gene or a controlling sequence, rather than to underlying causal reasons [19]. To overcome the biallelic problem and increase the resolution of candidate regions, the analysis of haplotypes can be employed, i.e. of the combination of co-inherited markers from polymorphic sites within a certain chromosome region. Exploring haplotypes in this way can take full advantage of ancient recombination events in order to identify the genetic loci underlying traits at a relatively high resolution [20].

The recently released wheat reference genome assembly (IWGSC Ref. Seq. v1.0), developed by the International Wheat Genome Sequencing Consortium (IWGSC) [21], allows a more accurate comparison of the chromosome regions associated with agronomic traits from independent research studies and helps accelerate cloning of further important genes for wheat breeding.

In this study, 102 Argentinean wheat cultivars were genotyped by using the 35k Axiom Wheat Breeder’s Genotyping Array [22]. The polymorphic SNPs were physically anchored to the wheat reference genome assembly [21] and the genetic structure of the collection was determined. Based on multi-environmental trial data, haplotype-based GWAS was conducted to identify chromosome regions affecting crop adaptation and the building of yields. Adaptation traits include heading date, a relevant trait since it will determine the environment to which the crop will be exposed during its reproductive stages, and plant height, which is the trait most associated with lodging in Argentinean cultivars [23]. Yield-related traits include thousand grain weight and number of grains per spike, currently called "numerical components of yield", which are the main factors proposed by [24] to analyze the differences in yield between cultivars. Additionally, [25] proposed considering the number of grains (GN) as the product between two variables currently known as "ecophysiological components": the spike dry weight at anthesis (SDWA) and the fruiting efficiency (FE, aka spike fertility index, ie the quotient between GNS and SDWA). Later [26] proposed the use of spike dry weight at harvest without grains (or chaff) as a substitute to SDWA. Then, the fruiting efficiency is calculated in this work as the grain number produced per unit of chaff (FEh). The FEh has shown a high association with the GN in both Argentinean [13, 27–29] and foreign [30] cultivars and moderately high heritability [12, 28, 29]. GWAS provides valuable information for a better understanding of the major genetic components involved in adaptation and yield components for bread wheat, with major implications for breeding programs aimed at increasing yield potential of new cultivars, in order to contribute to food security.

## Results

### SNP-based genetic structure of the Argentinean wheat collection

The genotyping of the 102 Argentinean hexaploid wheat cultivars collection yielded a total of 7972 polymorphic SNPs with MAF ≥ 10%. A set of 6486 polymorphic SNPs was genetically anchored to specific wheat chromosomes based on mapping information from five biparental populations described previously [22]. A second set of 909 additional SNPs were anchored to specific chromosomes using three local biparental populations and an additional 577 SNPs were anchored using the association method mentioned above (Additional file 1: Table S1). Of all the anchored SNPs, 3034 were on the A subgenome, 4010 on the B subgenome and the lowest number, 928 SNPs, on the D subgenome. To reveal the genetic relationships among the 102 Argentinean hexaploid wheat cultivars collection, we conducted a model-based approach using R STRUCTURE software [31]. A model-based approach is a cluster analysis that evaluates genetic similarity among genotypes without using prior information. For the analysis we used the 7972 SNPs anchored to the wheat genome described before. We detected four subpopulations in the collection reflecting the origin of the germplasm used by the main breeding programs in Argentina (Additional file 2: Table S2). A graphical representation of the STRUCTURE Q matrix was included (Additional file 3: Figure S1). Subpopulation 1 (Table 1) included only introduced cultivars of French origin (100%), with the Nidera, Syngenta and Sursem breeding companies exclusively represented in this group. Subpopulation 2 included most of the old cultivars (9 out of 12 released between 1930 and 1990), also known as traditional germplasm [32, 33] and all cultivars from the Don Mario breeding company released up until 2010. Subpopulation 3 included cultivars released by the Klein breeding company (46.15%) and, in lower frequency, cultivars belonging to the ACA, Buck, INTA and Relmó breeding companies, most of them with pedigrees including CIMMYT germplasm. Finally, subpopulation 4 included cultivars released by INTA (33.33%), Buck, Klein, ACA and Relmó, with most of them also having pedigrees including CIMMYT germplasm.

**Table 1:** Distribution of the 102 Argentinean bread wheat cultivars in the four subpopulations detected using a model-based approach

Subp.	Cultivar
1	Baguette 10; Baguette 17; Baguette 18; Baguette 19; Baguette 21; Baguette 30; Baguette 31; Baguette 9; Baguette P. 11; SY 100; SY 200; SY 300; SRN Nogal
2	ACA 201; ACA 320; Barletta 77; BIOINTA 1004; BIOINTA BONAERENSE 2001; BIOINTA 2004; Buck Chacarero; Buck Guapo; Buck Malevo; Buck Mangrullo; Buck Meteoro; Buck Napostá; Buck Norteño; Buck Pingo; Buck Ranquel; Buck Taita; Don Mario Arex; Don Mario Atlax; Don Mario Cronox; Don Mario Onix; Don Mario Themix; LE 2249; LE 2271; Klein 32; Klein Atlas; Klein Centauro; Klein Escorpión; Klein Impacto; Klein Proteo; Klein Rendidor; KleinTauro; Olaeta Artillero; Sinvalocho
3	ACA 223; ACA 903B; ACA 906; BIOINTA 1001; BIOINTA 3004; Buck Baqueano; Buck Huanchen; Buck Puelche; Klein Brujo; Klein Cacique; Klein Carpincho; Klein Castor; Klein Don Enrique; Klein Gavilán; Klein Gladiador; Klein Guerrero; Klein Nutria; Klein Tigre; Klein Yarará; Klein Zorro; LE 2333; LE 2341; ProINTA Gaucho; ProINTA Granar; ProINTA Guazú; ProINTA Oasis
4	55 CL 2; ACA 202; ACA 801; ACA 901; ACA 907; BIOINTA 1000; BIOINTA 1002; BIOINTA 1003; BIOINTA 1005; BIOINTA 1006; BIOINTA 3003; BIOINTA 3005; Buck 75 Aniversario; Buck AGP Fast; Buck Biguá; Buck Brasil; Buck Pronto; INIA Centinela; LE 2294; Klein Capricornio; Klein Chajá; Klein León; Klein Pantera; Klein Rayo; LE 2330; LE 2331; Marcos Juárez INTA; ProINTA Elite; ProINTA IslaVerde; Relmó Sirirí

### SNP and gene marker-based haplotype blocks construction and characterization of the Argentinean wheat cultivar collection

**Fig. 1:**
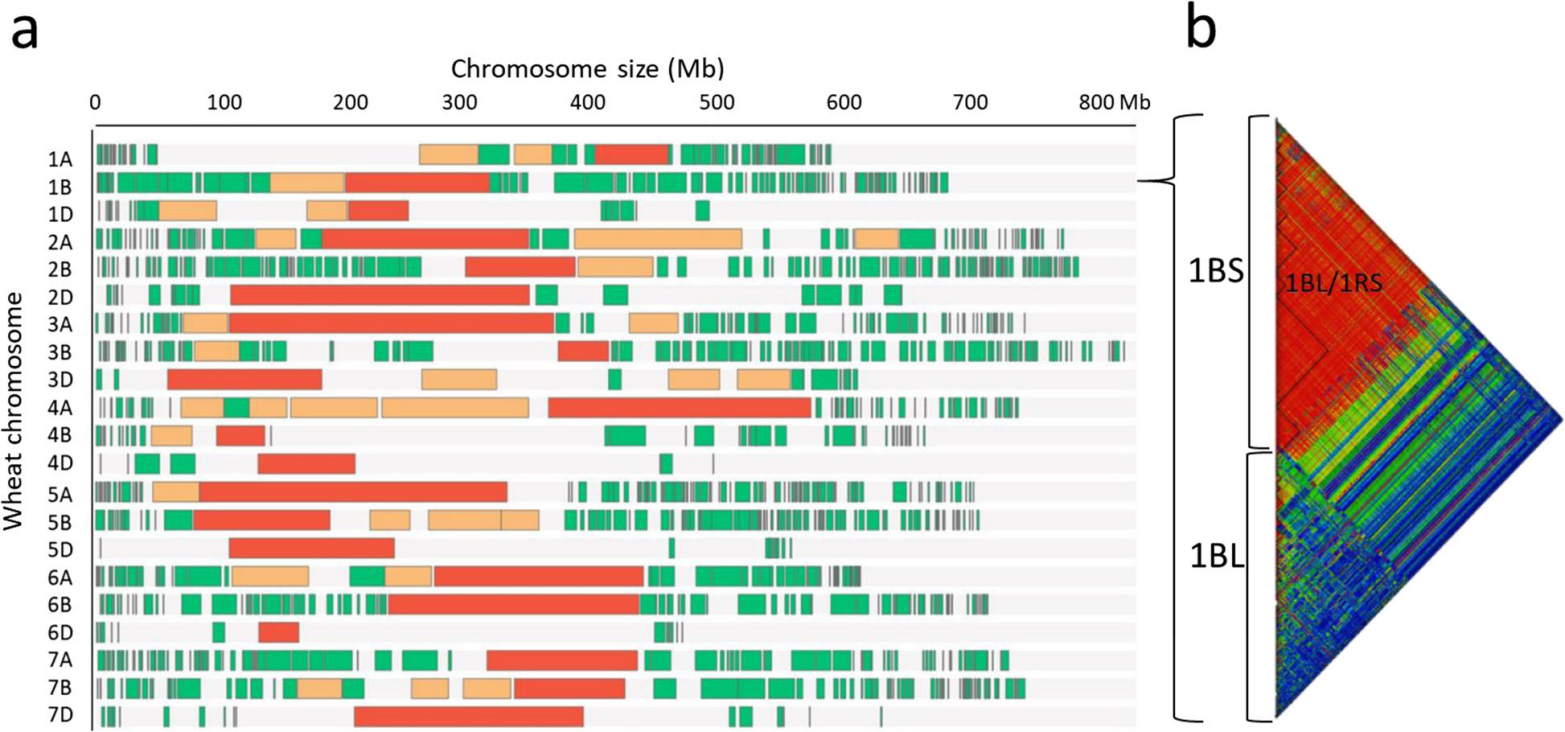
**a** HB sizes and position in each wheat chromosome based on IWGSC Ref Seq v1.0 coordinates. The red HBs indicate sizes >30 Mb, orange HBs sizes between 10 and 30 Mb and green HBs sizes <10 Mb. **b** Schematic representation of the LD (Linkage Disequilibrium) detected on chromosome 1B. In red, the high level of LD in the short arm of the chromosome due to the presence of the 1BL/1RS wheat/rye translocation

A detailed information about the haplotype-based map construction for the Argentinean wheat collection is found in the Table 2. A total of 1268 haplotype blocks (HBs) were built in our wheat cultivars collection, of which 518 were on the A subgenome, 641 on the B subgenome and 109 on the D subgenome. The 94% of the SNP and gene markers were involved in these HBs and only 495 SNP/gene-related markers were not included in any HB. The average block sizes were significantly larger on the D subgenome chromosomes than on the A or B subgenomes, with observed averages of 13.5, 6.7 and 4.8 Mb, respectively (Table 2). In general, within each chromosome, the largest HBs (> 30 Mb) were placed in the centromeric region, where recombination is lower, while the smallest HBs were located towards the telomeric regions, where the recombination rate and gene density are higher (Fig. 1a). The average distance between HBs on the D subgenome was 8 times larger than that for the B or A subgenomes, with an average of 16.1, 2.7 and 2.4 Mb, respectively. The maximum spacing between segments was also significantly higher in the D subgenome with respect to B and A, with average values of 106.6, 57.1 and 44.2 Mb, respectively (Table 2). An interesting case can be described for chromosome 1 B, where we observed several large HBs on the short arm, from HB 9 to 20 (HB average size 24 Mb in the region). A possible explanation is the presence of the 1BL/1RS wheat/rye translocation which is maintained at high frequency in Argentinean cultivars. The lack of recombination events within the translocation generates a highly conserved block involving almost the entire short arm of chromosome 1B (Fig. 1b) (Table 2).

**Table 2:** Detailed information about the haplotype-based map construction for the Argentinean wheat collection

Chr.	SNPs/	Haplotype	HB^3^	SNPs per HB		HB size^4^		HB spacing^4^	
SG^1^	GM^2^	count	count	Avg.	Max.	Avg.	Max.	Avg.	Max.
1A	431	244	66	6.2	26	4.5	59.4	3.4	125.4
1B	919	327	91	9.7	168	5.8	115.6	1.3	21.6
1D	253	74	22	10.8	90	8.5	48.8	10.0	115.8
2A	421	239	70	5.4	60	7.9	166.7	2.1	26.7
2B	709	459	127	5.2	40	3.8	88.8	1.8	35.9
2D	272	79	23	11.4	38	16.7	240.6	9.0	91.1
3A	389	268	75	4.8	22	6.7	261.7	2.3	28.6
3B	597	421	116	4.8	18	3.5	40.3	2.7	101.3
3D	115	56	15	7.4	21	21.8	124.5	16.9	80.7
4A	326	216	59	5.1	36	9.5	211.3	2.2	16.3
4B	253	141	42	5.3	26	4.7	39.1	6.6	162.4
4D	47	29	8	4.1	14	16.0	78.3	17.6	103.2
5A	544	340	99	5.3	38	4.6	247.9	2.1	49.8
5B	641	365	102	5.8	67	4.5	110.1	1.7	19.7
5D	110	36	11	9.3	28	13.7	133.7	21.6	106.2
6A	433	227	60	7.0	55	8.2	168.9	2.5	33.5
6B	532	300	86	5.8	55	5.6	202.1	2.0	21.1
6D	69	45	13	4.8	12	4.6	32.1	20.6	153.5
7A	509	330	89	5.3	34	5.2	121.4	2.3	28.8
7B	374	270	77	4.5	18	6.0	89.2	2.9	37.8
7D	80	50	17	4.2	11	13.2	185.0	16.9	95.7
A	436.1	266.3	74	5.6	38.7	6.7	176.8	2.4	44.2
B	575.0	326.1	92	5.9	56.0	4.8	97.9	2.7	57.1
D	82.1	52.7	16	7.5	30.6	13.5	120.4	16.1	106.6
Overall	436.9	269.5	60	6.3	41.6	8.3	131.7	7.1	69.3

^1^ SG = Subgenomes. ^2^ GM = Gene-related Markers. ^3^ HB = Haplotype Blocks. ^4^ The units are expressed in Mb based on the coordinates in the IWGSC Ref. Seq. v1.0 wheat genome assembly

### Broad sense heritability and correlations among traits

All evaluated traits showed high heritability: the crop adaptation traits HD and PH showed broad sense heritability values higher than 0.95 and the yield related traits FEh, TGW and GNS displayed values in all cases higher than 0.80 (Table 3).

**Table 3:** Broad sense heritability (*H*^2^) and variance components for the best linear unbiased predictors (BLUPs) of five traits across the tested environments

Trait	No. env.	$\sigma _{G}^{2}$	$\sigma _{E}^{2}$	$\sigma _{E}^{2}$	*H*^2^
FEh	10	74.69	232.24	139.43	0.84
TGW	10	5.24	20.19	12.63	0.81
GNS	10	17.43	28.59	34.77	0.83
HD	11	89.31	143.44	39.76	0.96
PH	11	79.94	109.18	26.52	0.97

$\sigma _{G}^{2}$ = genotypic variance; $\sigma _{E}^{2}$ = environmental variance; $\sigma _{E}^{2}$ = residual variance (here = $\sigma _{\mathit {GxE}}^{2}$ since BLUPs have a single replication per environment); *H*^2^ = broad sense heritability. All genotype and environment variances were signifcant at *P*<0.001

Regarding correlations between traits across the tested locations, the most consistent pattern was observed for FEh vs GNS, with positive correlations in most years at the Southern locations, Azul and Balcarce, and in all years at the Northern location Marcos Juárez (Table 4). The relationship between FEh vs TGW showed no significant correlations in all tested environments, except Azul 2014.

**Table 4:** Pearson’s correlation among traits in the different tested environments

	South locations				North location				
	Azul		Balcarce		Marcos Juárez				
Traits	13^1^	14	14	15	12	13	14	15	16
HDvsPH	-0.23*	ns	0.26*	0.32*	ns	ns	0.35**	ns	ns
HDvsFEh	0.23*	0.52***	ns	ns	ns	ns	ns	ns	-0.31*
HDvsTGW	ns	-0.42***	ns	ns	ns	ns	ns	-0.47***	-0.34**
HDvsGNS	ns	0.44***	0.26*	ns	ns	-0.31*	ns	-0.41***	-0.44***
PHvsFEh	-0.28*	ns	-0.32*	-0.35**	ns	ns	ns	ns	ns
PHvsTGW	ns	0.24*	ns	ns	0.48***	0.37**	0.40***	0.39***	0.43***
PHvsGNS	-0.23*	ns	-0.38***	-0.31*	0.21*	ns	ns	ns	ns
FEhvsTGW	ns	-0.67***	ns	ns	ns	ns	ns	ns	ns
FEhvsGNS	ns	0.47***	0.35**	0.24*	0.58***	0.31*	0.38**	0.36**	0.58***
TGWvsGNS	0.22*	-0.21*	ns	ns	ns	ns	0.25*	0.48***	0.43***

^1^ years after 2000; ns = not significant, * *P* <0.05, ** *P* <0.001, *** *P* <0.0001. HD, heading date; PH, plant height; FEh, fruiting efficiency at harvest; TGW, thousand grain weight; GNS, grains number per spike

The PH showed a positive correlation with TGW in Azul (one year) and in all years in Marcos Juárez with higher significant values. On the other hand, the PH showed negative correlations with GNS in most years at the Southern locations, contrasting with Marcos Juárez with no significant correlation in most tested years. A similar pattern can be described for PH vs FEh, with negative correlations in most years at the Southern locations, contrasting with Marcos Juárez with no significant correlations in all years (Table 4).

The HD showed a positive correlation with GNS in the Southern locations (2 years), contrasting with Marcos Juárez with negative correlations in three years. The HD also showed negative and highly significant correlations with TGW in Azul (1 year) and Marcos Juárez (2 years). A positive correlation between HD and FEh was observed in Azul (2 years), differing from Marcos Juárez with no significant correlations in most tested years. The HD vs PH showed contrasting correlations at the Southern locations and no significant correlations in most years in Marcos Juárez. The TGW vs GNS correlation showed positive values in Marcos Juárez (3 years), with no significant correlations in most Southern environments (Table 4).

Contrasting correlations observed across the Southern and Northern locations for PH vs GNS, PH vs FEh, HD vs GNS, HD vs FEh and, to a lesser extent, PH vs TGW, might suggest different strategies are involved in building site-specific yield components performance: genotypes adapted to the Northern latitudes would be favored by taller plants improving TGW, but affected by late flowering penalyzing TGW and GNS, whereas genotypes adapted to Southern latitudes would be favored by late flowering improving GNS and FEh, but affected by taller plants penalyzing FEh. Interestingly, independently of the environment, genotypes with high FEh would favor GNS, one of the most important yield components.

### Haplotype-based gWAS analysis

#### Fruiting efficiency at harvest (FEh):

We identified 17 haplotypes/markers associated with FEh of which one belongs to chromosome 1A, seven to 2A, one to 3B, two to 4A, two to 5A, three to 6A and one to 7A (Table 5; Additional file 6: Table S5). Eleven haplotypes associated with FEh were significant in at least one year in each of the three locations, i.e. Southern (Azul and Balcarce) and Northern (Marcos Juárez). Five haplotypes were significant exclusively in Southern locations and only the SNP AX-94874921 located on chromosome 2A (707.1Mb) showed significant association exclusively in Marcos Juárez.

**Table 5:** GWAS results for FEh, TGW, GNS, HD and PH. Information of haplotypes/SNPs physical location, frequency in the collection and effect direction, *P* values in each environment and the colocated traits

		Ref. Seq. v1.0 (Mb)			Freq.	Azul		Balcarce			Marcos Juárez						
Trait	Haplotype/SNP^1^	Chr.	Start	End	Effect^2^	13^3^	14	13	14	15	11	12	13	14	15	16	Coloc.
FEh	Chr1A-B32-Hap4	1A	482.3	487.0	6+	*	ns	*	*	**	-	ns	ns	ns	ns	ns	
	Chr2A-B19-Hap3	2A	70.2	72.9	12+	*	ns	*	*	ns	-	**	ns	*	ns	ns	
	Chr2A-B23-Hap1	2A	83.8	84.5	52-	*	*	*	*	ns	-	*	ns	ns	*	*	
	Chr2A-B26-Hap3	2A	102.0	102.7	36+	*	**	**	*	ns	-	ns	ns	ns	ns	*	
	Chr2A-B30-Hap4	2A	165.8	182.1	7+	**	ns	*	*	ns	-	ns	*	ns	ns	ns	
	Chr2A-B49-Hap2	2A	704.8	705.8	15+	*	*	*	**	*	-	ns	ns	ns	ns	ns	GNS
	AX-94874921	2A	707.1	-	16+	ns	ns	ns	ns	ns	-	**	ns	*	*	*	
	Chr2A-B55-Hap1	2A	716.8	718.9	12+	*	***	**	*	*	-	ns	ns	ns	ns	ns	
	Chr3B-B14-Hap4	3B	23.0	24.0	6+	ns	ns	**	*	*	-	**	ns	ns	*	ns	
	AX-94869767	4A	606.4	-	18+	*	ns	*	*	ns	-	*	*	ns	*	*	
	Chr4A-B35-Hap3	4A	625.7	625.9	23+	**	*	**	*	*	-	ns	ns	ns	ns	ns	
	Chr5A-B33-Hap4	5A	445.2	445.2	7+	**	*	*	*	ns	-	ns	ns	ns	ns	ns	PH
	Chr5A-B43-Hap1	5A	476.4	476.7	15+	*	ns	*	*	ns	-	ns	**	ns	*	ns	
	Chr6A-B24-Hap2	6A	205.1	233.3	17+	***	*	*	ns	ns	-	ns	ns	ns	ns	*	TGW
	Chr6A-B28-Hap4	6A	448.7	454.6	9-	*	*	*	**	*	-	*	ns	ns	ns	ns	
	Chr6A-B29-Hap2	6A	455.6	467.0	25+	***	ns	*	*	ns	-	ns	ns	*	ns	*	
	Chr7A-B36-Hap2	7A	119.0	126.0	10+	*	ns	*	**	*	-	ns	ns	ns	ns	*	
TGW	Chr2D-B5-Hap3	2D	17.6	18.2	7-	*	*	ns	ns	ns	-	ns	ns	*	*	**	
	Chr3A-B12-Hap3	3A	46.7	52.6	5-	ns	*	ns	*	*	-	**	*	*	ns	ns	
	AX-95257035	3A	686.8	-	11+	ns	ns	ns	ns	ns	-	*	ns	***	**	*	
	Chr3B-B111-Hap4	3B	817.4	817.8	11+	ns	ns	ns	*	ns	-	**	ns	*	*	ns	PH
	AX-94459169	6A	0.6	-	30+	ns	**	*	ns	ns	-	ns	*	***	***	**	
	Chr6A-B20-Hap3	6A	63.8	74.0	21-	ns	ns	*	ns	ns	-	ns	ns	*	**	*	
	Chr6A-B21-Hap3	6A	74.5	101.3	20-	ns	ns	*	ns	ns	-	ns	ns	*	**	*	
	Chr6A-B24-Hap2	6A	205.1	233.3	17-	ns	ns	**	ns	ns	-	*	ns	*	***	*	FEh
	Chr7B-B38-Hap3	7B	626.1	627.6	5+	ns	***	**	ns	ns	-	ns	*	*	ns	**	
	Chr7B-B60-Hap2	7B	702.3	703.7	27+	ns	ns	ns	*	ns	-	*	*	**	*	*	
	Chr7B-B60-Hap3	7B	702.3	703.7	15+	ns	ns	*	ns	ns	-	*	*	**	**	**	
GNS	Chr2A-B49-Hap2	2A	704.8	705.8	15+	*	ns	*	**	**	-	ns	ns	*	ns	ns	FEh
	Chr4A-B19-Hap5	4A	231.0	349.3	13+	*	**	ns	ns	ns	-	**	*	*	ns	*	
HD	Chr1B-B76-Hap2	1B	643.1	645.7	35+	*	**	ns	ns	ns	**	*	*	ns	*	*	
	Chr1B-B84-Hap3	1B	667.2	667.8	17-	ns	ns	**	**	*	ns	*	*	*	ns	ns	
	Chr1B-B89-Hap4	1B	676.8	678.0	6-	ns	ns	*	**	*	*	*	ns	*	*	ns	
	Chr2B-B40-Hap4	2B	162.9	166.9	10+	*	**	ns	ns	ns	ns	*	ns	*	*	ns	
	Chr2B-B62-Hap1	2B	564.2	569.0	10+	*	**	ns	ns	ns	*	ns	ns	ns	*	ns	
	*Ppd-D1* sensitive	2D	34	-	42+	*	*	*	*	*	**	***	**	***	***	**	
	Chr3A-B31-Hap1	3A	504.8	507.5	59-	ns	ns	*	ns	*	**	*	*	*	*	*	
	Chr3A-B49-Hap3	3A	631.2	633.1	12-	**	ns	*	*	ns	*	*	ns	*	**	*	
	Chr3A-B59-Hap3	3A	695.5	695.7	19+	ns	*	ns	ns	ns	**	*	**	*	*	ns	
	Chr3A-B60-Hap4	3A	699.4	700.6	10+	ns	**	ns	ns	ns	**	*	***	**	*	ns	
	Chr3D-B7-Hap1	3D	518.2	560.1	54-	*	*	ns	ns	ns	*	*	**	*	*	*	
	Chr5B-B75-Hap4	5B	597.2	601.4	11-	*	ns	ns	ns	ns	*	*	ns	*	**	ns	
	AX-94610041	6A	13.8	-	46+	*	*	**	*	*	**	ns	*	*	*	ns	
	Chr6A-B43-Hap1	6A	591.7	592.5	61-	ns	**	ns	ns	ns	*	*	**	*	*	*	
	Chr6B-B26-Hap3	6B	157.6	157.8	11+	*	**	ns	ns	ns	ns	ns	ns	*	*	**	PH
	Chr7A-B24-Hap2	7A	68.0	70.2	8+	ns	ns	*	**	*	*	ns	**	*	*	ns	
PH	Chr1A-B38-Hap3	1A	508.6	510.4	16+	*	**	ns	ns	ns	*	ns	ns	*	ns	ns	
	Chr1B-B17-Hap4	1B	121.0	124.0	5+	*	*	*	**	*	***	*	*	*	*	**	
	AX-94510167	1B	548.7	-	21+	ns	*	ns	**	*	**	ns	ns	*	ns	ns	
	Chr1B-B54-Hap1	1B	580.5	581.2	43-	*	**	ns	*	*	*	ns	ns	***	***	ns	
	*Lr10*	1D	8.6	-	10+	*	*	**	ns	*	ns	*	ns	*	*	ns	
	Chr1D-B5-Hap4	1D	11.6	11.9	19-	*	**	ns	*	*	*	ns	*	*	*	*	
	Chr1D-B10-Hap4	1D	31.1	32.5	10+	*	ns	***	*	ns	*	*	ns	ns	ns	ns	
	Chr2A-B39-Hap1	2A	611.2	611.9	70-	**	ns	**	ns	ns	*	ns	*	ns	ns	*	
	Chr2A-B48-Hap3	2A	701.0	702.1	15-	*	***	*	ns	*	*	ns	ns	*	*	ns	
	Chr2B-B56-Hap4	2B	469.2	476.6	6+	ns	*	**	*	ns	*	ns	*	ns	**	*	
	Chr2B-B79-Hap3	2B	664.2	664.9	5+	*	ns	**	*	ns	**	ns	*	ns	**	*	
	AX-94687989	2B	702.2	-	52+	ns	**	ns	ns	ns	ns	*	ns	**	*	ns	
	Chr2B-B97-Hap1	2B	719.5	719.5	38-	ns	**	ns	ns	ns	ns	*	ns	**	*	*	
	Chr2B-B114-Hap4	2B	762.0	763.9	8+	**	*	*	ns	*	ns	ns	ns	*	ns	ns	
	AX-94585596	2B	776.4	-	15+	*	**	ns	ns	*	**	*	*	*	ns	ns	
	AX-94624485	2B	776.8	-	10+	*	*	*	ns	ns	**	*	*	ns	ns	ns	
	Chr2B-B122-Hap3	2B	777.1	777.2	6+	*	*	*	ns	*	***	**	*	*	*	ns	
	Chr3A-B22-Hap2	3A	391.5	393.4	53+	*	*	ns	*	ns	*	**	*	*	ns	*	
	AX-95090222	3A	700.6	-	18+	**	ns	ns	*	ns	ns	**	ns	*	ns	ns	
	AX-95083205	3A	743.9	-	46+	**	*	ns	ns	*	ns	ns	*	*	ns	ns	
	Chr3B-B6-Hap1	3B	16.4	17.1	65-	*	*	*	ns	*	ns	*	*	**	**	*	
	Chr3B-B55-Hap4	3B	511.1	513.6	6-	**	*	ns	ns	*	ns	ns	ns	ns	*	*	
	Chr3B-B60-Hap6	3B	541.5	544.5	5+	*	ns	*	*	ns	**	*	*	*	*	ns	
	Chr3B-B109-Hap2	3B	796.0	799.1	9-	*	**	*	ns	ns	ns	ns	ns	ns	*	*	
	Chr3B-B111-Hap4	3B	817.4	817.8	11+	***	ns	*	*	**	***	*	**	*	*	*	TGW
	Chr4A-B57-Hap6	4A	738.7	739.7	9-	*	ns	ns	**	ns	**	ns	ns	ns	*	ns	
	Chr4B-B28-Hap5	4B	612.2	613.2	6+	*	**	*	*	*	*	*	*	*	*	ns	
	Chr4B-B36-Hap2	4B	650.7	650.7	57-	**	*	*	*	ns	*	**	**	ns	*	ns	
	Chr5A-B16-Hap4	5A	33.0	33.2	5+	*	*	*	*	*	**	**	ns	ns	ns	ns	
	Chr5A-B33-Hap4	5A	445.2	445.2	7-	ns	***	*	ns	ns	*	ns	ns	ns	*	*	FEh
	Chr5A-B54-Hap3	5A	516.1	527.9	5+	ns	*	*	**	**	***	ns	ns	*	*	*	
	Chr5A-B82-Hap2	5A	611.6	613.5	10+	*	ns	*	ns	ns	*	***	ns	*	ns	*	
	Chr5B-B26-Hap2	5B	430.7	430.7	17+	**	*	ns	*	*	*	*	ns	ns	ns	*	
	AX-94550178	5B	455.7	-	46+	ns	ns	***	*	*	*	ns	ns	*	ns	ns	
	Chr5B-B92-Hap4	5B	683.5	684.0	5+	*	ns	*	*	ns	**	*	*	*	ns	**	
	Chr6A-B2-Hap5	6A	0.6	0.8	5+	*	ns	**	**	*	ns	ns	ns	*	*	ns	
	Chr6A-B4-Hap2	6A	2.2	2.7	26+	*	ns	*	*	**	ns	*	ns	ns	*	ns	
	Chr6A-B4-Hap3	6A	2.2	2.7	17-	**	*	*	ns	*	ns	*	ns	*	**	*	
	Chr6A-B16-Hap1	6A	48.6	49.8	57+	ns	ns	ns	*	ns	*	*	ns	**	*	**	
	Chr6A-B17-Hap3	6A	50.3	51.4	33-	ns	*	*	*	ns	**	ns	*	*	**	ns	
	Chr6A-B54-Hap5	6A	610.0	610.2	6+	ns	ns	*	*	ns	***	ns	**	ns	ns	ns	
	Chr6B-B7-Hap2	6B	19.8	20.7	82-	**	***	**	*	ns	*	***	*	*	*	*	
	Chr6B-B20-Hap4	6B	124.4	126.1	6+	*	ns	ns	ns	ns	**	*	*	ns	*	*	
	Chr6B-B25-Hap4	6B	151.4	156.4	25-	*	**	*	*	**	ns	ns	ns	*	*	ns	
	Chr6B-B26-Hap3	6B	157.6	157.8	11-	*	***	ns	*	ns	*	*	*	ns	*	***	HD
	Chr6B-B31-Hap2	6B	195.3	197.8	5+	*	ns	**	**	*	**	ns	ns	*	*	*	
	AX-94943227	6B	234.8	-	17+	**	***	**	*	*	*	***	*	*	*	*	
	Chr6B-B47-Hap3	6B	480.4	491.6	24-	ns	ns	ns	*	**	ns	ns	ns	*	ns	*	
	AX-94476474	7A	561.6	-	14+	**	*	*	ns	ns	ns	ns	**	*	*	ns	
	Chr7B-B14-Hap4	7B	59.6	61.6	22-	***	ns	ns	ns	*	ns	*	ns	ns	*	ns	
	Chr7B-B76-Hap3	7B	743.5	743.6	10-	**	***	*	*	ns	*	ns	*	**	**	*	

^1^ Information about SNPs ID and/or gene molecular marker that were involved in the haplotypes was detailed in the Additional file 6: Table S5. ^2^ Freq. = Number of genotypes that present the SNP / haplotype in the collection. Effect (+) the SNP / haplotype increases the phenotypic value and (-) reduces the phenotypic value. ^3^ Years after 2000. Only SNPs that passed selection criteria in GWAS are presented in the table. ns = not significant, * *P* <0.05, ** *P* <0.001, *** *P* <0.0001, "–" = not available

**Fig. 2:**
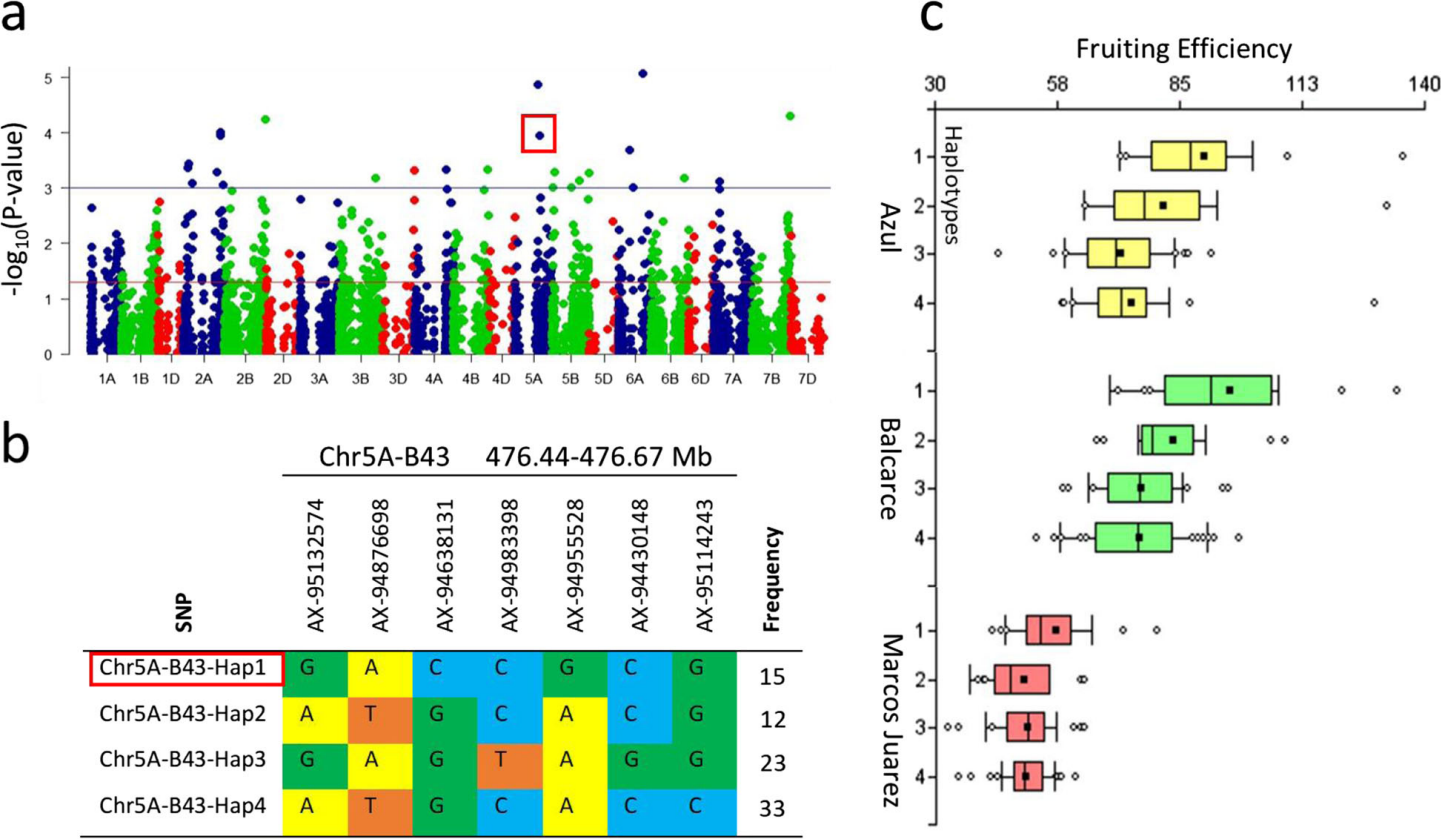
**a** Manhattan plot on 21 wheat chromosomes for fruiting efficiency at harvest (FEh) in Balcarce 2013. Red line represents the GWAS threshold of *P* <0.05 = −*log*_10_ (*P*-value) = 1.3 and blue line represents the GWAS threshold of *P* <0.001 = −*log*_10_ (*P*-value) = 3. Red square highlights the Chr5A-B43-Hap1 associated with FEh in five of ten tested environments. **b** Haplotype block based on seven SNP markers located between 476.44 and 476.67Mb on chromosome 5A, named Chr5A-B43. Four different haplotype variants (Hap1–Hap4) are present at different frequencies in the analyzed population. Red rectangle highlights the Chr5A-B43-Hap1 associated with FEh. **c** Boxplots indicate the phenotype values corresponding to the four different haplotype groups in the three evaluated locations. Hap1 was associated significantly high FEh in all locations

An example of a haplotype with significant association with FEh across Southern and Northern locations is Chr5A-B43-Hap1, located on chromosome 5A (476.4-476.7 Mb) (Fig. 2a). The HB Chr5A-B43 is composed of seven SNPs, and the significantly associated haplotype Hap1.The Chr5A-B43-Hap1 was detected in 16 out of 102 evaluated cultivars (Fig. 2b). The presence of Chr5A-B43-Hap1 significantly increased FEh in the three tested locations, albeit with different intensities, with a stronger effect in Southern locations (Fig. 2c).

Three haplotypes significantly associated with FEh were detected as colocating with additional traits: haplotype Chr2A-B49-Hap2 on 2A (704.8-705.8 Mb) was colocated with GNS, haplotype Chr5A-B33-Hap4 on chromosome 5A (445.2-445.2 Mb) with PH and Chr6A-B24-Hap2 on chromosome 6A (205.1-233.3 Mb) with TGW. The correlations for the first two haplotypes were significant only in Southern locations (Azul and Balcarce), while the third was significant in all locations. In the case of Chr2A-B49-Hap2, the correlation with GNS was positive, as shown in Table 5, and the cultivars with the haplotype for higher FEh also presented higher GNS. In the case of Chr5A-B33-Hap4, the correlation with PH was negative (Table 5) and cultivars with the haplotype for higher FEh values showed lower values for PH. Finally, for Chr6A-B24-Hap2, the correlation was also negative (Table 5): cultivars with the haplotype for higher FEh values showed lower values for TGW.

#### Thousand grain weight (TGW):

We identified 11 haplotypes/markers associated with TGW, of which one belongs to chromosome 2D, two to 3A, one to 3B, four to 6A and three to 7B (Table 5). The SNP AX-95257035 located on chromosome 3A (686.8 Mb) was significantly associated with TGW only in Marcos Juárez in four of five tested years. The remaining ten haplotypes/markers showed significant associations with TGW across Southern and Northern locations with, in general terms, higher significance values at the Northern location (Table 5).

Two haplotypes significantly associated with TGW and colocating with other traits were detected. The Chr3B-B111-246 Hap4 on chromosome 3B (817.4-817.8 Mb) colocated with PH, with the previously described Chr6A-B24-247 Hap2 on chromosome 6A (205.1-233.3Mb) colocating with FEh. In the case of Chr3B-B111-Hap4, the correlation with PH was positive, as cultivars carrying the haplotype for higher TGW also expressed higher PH (Table 5).

#### grain number per spike (GNS):

We identified two haplotypes associated with GNS, one belonging to chromosome 2A and the second to 4A (Table 5). The Chr2A-B49-Hap2 was significantly associated across all locations and Chr4A-B19-Hap5 was significantly associated in Azul and Marcos Juárez, though with no significant effect in Balcarce. The haplotype Chr2A-B49-Hap2 colocated with FEh and its effect was previously described (Table 5).

#### Heading date (HD):

A total of 16 haplotypes/markers showed significant associations with HD. The regions were distributed as follows: three on chromosome 1B, two on 2B, one on 2D, four on 3A, one on 3D, one on 5B, two on 6A, one on 6B and one on chromosome 7A. For this trait, it is important to highlight the consistent effect of the functional marker for the *Ppd-D1* gene on chromosome 2D, which showed significant associations over the three tested locations during in all evaluated years. The highest associations were detected in Marcos Juárez with latitude 5^∘^ lower than Southern locations (Azul and Balcarce). Cultivars carrying the *Ppd-D1* sensitive allele (42 of the 102 cultivars tested) delayed flowering in all environments. Also it is important to mention that all haplotypes affecting HD showed significant associations at the Northern location in two or more years, contrasting with Southern locations Azul, showing three haplotypes with no significant effects on HD, and Balcarce, with nine haplotypes in the same situation. Additionally, we identified one haplotype colocated with PH, Chr6B-B26-Hap3 (157.6-157.8Mb), that increased HD values and negatively affected PH (Table 5).

#### Plant height (PH):

In the haplotype-based GWAS, 51 chromosome regions were associated with this trait. Among these, one was located on chromosome 1A, three on 1B, three on 1D, two on 2A, eight on 2B, three on 3A, five on 3B, one on 4A, two on 4B, four on 5A, three on 5B, six on 6A, seven on 6B, one on 7A and two on chromosome 7B. Forty-six haplotypes/markers showed significant associations across all locations, including haplotype Chr1B-B17-Hap4 and SNP AX-94943227 on chromosome 6B that showed consistent significant effects in all tested locations and years. Three haplotypes significantly associated with PH colocated with traits FEh, TGW and HD, with contributions previously described (Table 5).

## Discussion

### Genetic structure and haplotype characterization

In this work, results of a model-based cluster analysis differentiated four subpopulations, whereas previously [32], using the same 102 cultivars and a small number of markers associated with genes of agronomic interest and neutral markers (SSRs and ISBPs), detected three subpopulations, which nonetheless and as expected overlapped with the four identified here. In general terms, subpopulations 1 and 2 in our study matched with subpopulations 1 and 3 of the previous work, in including introductions of European origin and traditional germplasm, respectively. Subpopulations 3 and 4 in our study matched subpopulation 2 of the previous work, all including in most cases germplasm with pedigrees of CIMMYT origin. Differences between subpopulations 3 and 4 may be related to the presence of the 1BL/1RS wheat-rye translocation, as 24/26 cultivars in subpopulation 3 carry this, whereas no cultivar is in a similar situation in subpopulation 4 (data not shown). This translocation has been widely used in breeding to achieve resistance to several pathogens and insects, to broaden adaptation and to increase yield [34]. Argentina is no exception, as modern cultivars carrying 1BL/1RS have shown: Klein Gladiador, Klein Nutria, Klein Yarará, LE 2333, LE 2341 and ACA 906, released between 2009 and 2010, confirm the consistent contribution of the translocation to biotic and abiotic stress tolerance. In spite of these advantages, special attention should be given to the use of 1BL/1RS, due to its detrimental effects on gluten strength and bread-making quality, albeit that they vary depending upon the genetic background [35].

Wheat has been exposed to intense artificial and natural selection since its domestication, resulting in large HBs as observed in elite germplasm [18, 36]. This issue can be observed in our work with the just mentioned presence of the 1BL/1RS wheat-rye translocation (Fig. 1b). In any case, we must also take into account the position of the HB in the chromosome, as we see in Fig. 1a, where large haplotypes (> 30 Mb) are formed in centromeric and pericentromeric regions of each chromosome. As previously reported in the work of [37], these regions can be a challenge when introducing allelic variants of interest in the region and reducing the size of the HBs.

### GWAS analysis

#### Yield potential related traits:

Fruiting efficiency has been proposed as a promising trait for increasing yield potential in wheat [12, 13, 28, 38, 39]. We detected 15 significant haplotypes/markers with positive effects on FEh and only two negatives (Table 5). The strongest genetic associations for FEh were detected in the Southern locations Azul and Balcarce, with lower associations observed in Marcos Juárez. These results imply FEh could provide an avenue to increase yield in regions where yield potential is high, as in the case of the Southern locations in our study.

In a previous study, a positive correlation between FEh and grain number was detected [13, 39]. Here, we detected a haplotype Chr2A-B49-Hap2 (704.8-705.8Mb) on chromosome 2A, that positively modifies both the traits FEh and GNS (Table 5). In a further study, [40] detected a SNP associated with FE and grains per spikelet (RFL_Contig3780_64), located at 676.24 Mb on chromosome 2A. They proposed CONSTANS 4 (CO4) and TaVrs1 genes as candidates to explain the variation detected. However, based upon the IWGSC Ref Seq. V1.0, CO4 is located at 594.58 Mb (81.66 Mb proximal to RFL_Contig3780_64). Our haplotype Chr2A-B49-Hap2 is located 28.56 Mb distal from RFL_Contig3780_64. The physical distance between the Chr2A-B49-Hap2 and CO4 is estimated as 110.22 Mb, and, taking into account the critical value for the decay of linkage desequilibrium (LD) by 0.1 is estimated as an average of 50 Mb for the wheat genome [41], would discard CO4 as a candidate for the FEh QTL detected at Chr2A-B49-Hap2. On the other hand, the gene *TaVrs1*, also called *GNI-A1*, was recently cloned and characterized by [42]; unfortunately the *GNI-A1* gene is not assembled on IWGSC Ref Seq. V1.0 chromosome 2A and its location cannot be compared to the Chr2A-B49-Hap2 haplotype.

In contrast, a negative relationship between FEh and TGW has also been described [28, 39]. In the current work, we detected haplotype Chr6A-B24-Hap2 (205.1-233.3Mb) that increases FEh values, but penalizes TGW.

Little is known about the relationship between FEh and PH. We detected a haplotype on chromosome 5A, Chr5A-B33-Hap4 (445.2 Mb) that associates with both traits, but in opposite directions.

Another relevant trait in the determination of yield potential is grain weight. We detected six significant haplotypes/markers with positive effects and five with negative effects on TGW (Table 5). This list includes four haplotypes on chromosome 6A associated with TGW, one of them Chr6A-B24-Hap2 (205.1-233.3Mb). This haplotype, negatively associated with TGW and positively associated with FEh, was located 4.5Mb proximal to the gene *TaGW2-A1*, which is located at 237.75Mb on chromosome 6A and has been shown to be related to grain weight [43]. Non-functional mutants for this gene increase the weight and size of wheat grains [44]. Shared function and physical location would turn *TaGW2-A1* into a promising candidate gene for Chr6A-B24-Hap2. On the other hand, the gene *TaGS5-3A* located at 176.55Mb on chromosome 3A has also been associated with larger grain size and higher thousand grain weight [45]. We found one haplotype and one SNP associated with TGW on chromosome 3A (Table 5), but they were located too far away from *TaGS5-3A* (123 and 510Mb apart, respectively) to be considered as a gene candidate. In the opposite direction to that observed for FEh, the strongest genetic associations for TGW were detected in the Northern location Marcos Juárez, promoting TGW as an interesting trait to increase yield for those latitudes.

For GNS, [46] used GWAS to detect a region located at 691.22Mb on chromosome 2A that significantly affects the number of grains per spike. We detected the haplotype Chr2A-B49-Hap2, located at 704.8-705.8 Mb, that positively affects GNS, only 13 Mb distal from the [46] region.

#### Crop adaptation related traits:

For HD, we found that the *Ppd-D1* gene marker [47] is the strongest associated with the trait, especially in the location Marcos Juárez, situated at the lowest latitude in our study. These results agree with those obtained by [33], where the *Ppd-D1* gene was found to be the main determinant of life cycle in Argentinean wheat cultivars.

On chromosome 1B, we detected three haplotypes associated with HD. [48] proposed the gene *TaFT-B3* (581.4Mb) as a HD modifier for short days on this chromosome. Our nearest significant haplotype, Chr1B-B76-Hap2 (Table 5), is located 61.7 Mb distal from *TaFT-B3*, being slightly farther than the critical value of 0.1 for LD decay estimated as 50 Mb [41].

On the other hand, [49] showed that loss of function mutants of the PHYTOCLOCK 1 (*WPCL1*) gene, located at 740.1Mb on chromosome 3A, were associated with extra-early flowering time. We detected four haplotypes on chromosome 3A associated with HD. Our nearest significant haplotype, Chr3A-B-Hap4 (Table 5), is located 39.5Mb proximal to *WPCL1* positioning WPCL1 as candidate for the Chr3A-B-Hap4 HD association.

Finally, no significant associations were detected for HD with the genes *Vrn-A1* (5A) [50], *Vrn-B1* (5B), *Vrn-D1* (5D) [51] and *Ppd-B1* (2B) [52], indicating that this set of genes did not appear to affect heading time in our cultivar collection. The most likely explanation is that may come from the field conditions where the collection was evaluated, with satisfied vernalization requirements, or potentially high epistatic interactions among vernalization genes, causing difficulties in the detection of minor effects on flowering time.

In the case of PH, we did not find significant associations between the trait and the molecular markers for the “green revolution” dwarfism genes *Rht-B1* and *Rht-D1*, described by [53]. Although the collection presented a wide variation in PH, it should be mentioned that the wheat collection used in our study is mainly composed of semi-dwarf elite germplasm and that the *Rht-B1* and *Rht-D1* genes are balanced.

Within the 51 significant associations detected, the chromosome 1B haplotype Chr1B-B17-Hap4 and the chromosome 6B SNP AX-94943227 (234.8 Mb) were particularly noteworthy, since they were significant across the three locations in all analyzed years. To our knowledge, there are no plant height related genes described on these chromosomes, which suggest these consistent PH-HB/SNP associations are promising targets for further gene cloning projects.

We detected four haplotypes on chromosome 5A associated with PH. In a mapping work [54] located the GA-responsive *Rht* genes *Rht9* and *Rht12* on chromosome arm 5AL, where *Rht9* was linked to the SSR barc151 (558.34 Mb) and *Rht12* was located 5.4cM from the SSR Xgwm291 (698.19 Mb). Our haplotype Chr5A-B54-Hap3 mapped at 516.1-527.9 Mb might be associated with *Rht9* linked to SSR barc151 at 558.34Mb. However, such an association with *Rht12* has to be discarded in our study, since additional PH haplotypes are placed in genetic regions distant to this PH gene.

On chromosome 6A, we detected six haplotypes associated with PH; in particular, the haplotype Chr6A-B54-Hap5 (610.0 Mb) was significantly associated with PH in the three locations and in all evaluation years, except Balcarce 2015. However, none of the associated haplotypes was located in physical positions close to any of the known PH genes on 6A, namely *Rht25* (144.0-148.3 Mb) detected by [55] and *Rht18/Rht14/Rht24*(413.73 Mb) described previously [56].

It is important to highlight that small association panels have been shown to increase both type 1 and type 2 error rates, failing to detect true associations while also generating a higher rate of false positive associations [57]. In this work, we used a small association panel (102 cultivars), but we used a conservative approach for GWAS, in order to reduce spurious associations. However, we may be failing to detect genomic regions that have low rates of explanation of phenotypic values or are found in low frequency in the panel. It is recommended to validate the associations reported in independent populations before being used in wheat breeding programs.

## Conclusion

The genetic structure of the Argentinean hexaploid wheat collection was determined by the use of SNP markers, in which a strong relationship was displayed between the subpopulations obtained and the germplasm origin used by the main breeding programs in the country. Based on SNP and gene-related markers physically anchored to the IWGSC Ref Seq v1.0, a haplotype map was constructed, allowing the detection of highly conserved and selected regions like the 1BL/1RS translocation. A GWAS detected ninety-seven chromosome region associated with yield components and adaptation. In the case of yield components, we highlight regions on chromosomes 1A, 2A, 3B, 4A, 5A, 6A and 7A associated with FEh, particularly at higher yield potential locations. For adaptation, the most relevant effect on HD was the *Ppd-D1* gene on chromosome 2D, being the main determinant in the variations in the life cycle of the Argentinean wheats. The use of the IWGSC Ref Seq v1.0 allowed us to precisely compare all detected associated regions with genes and QTLs reported in previous studies.

## Methods

### Plant material

A previously described [32] set of 102 bread wheat cultivars that includes old (such as cv. Barletta 77 released in 1927) and recent (up to 2010) commercial cultivars were selected from the main wheat breeding companies in Argentina and used for the haplotype block construction and GWAS analysis. Seed stocks were kindly provided by the Instituto Nacional de Tecnología Agropecuaria (INTA) Experimental Station Marcos Juárez, Wheat Germplasm Bank (Marcos Juárez, Argentina).

### Genotypic data

The population was genotyped at the Instituto de Genética Veterinaria (IGEVET) Genotyping Laboratory, La Plata, Argentina, using the 35k Axiom Wheat Breeder’s Genotyping Array [22]. Information about the 35k SNPs Axiom array is publicly available in CerealsDB^1^. SNPs with minor allele frequency (MAF) <0.10 and/or with more than 10% of missing data were discarded. The polymorphic SNPs were genetically anchored to specific wheat chromosomes according to previously described map information [22]. In a second step, linkage information from three biparental mapping populations was used to anchor new markers to wheat chromosomes. Additionally, several SNPs were anchored by transforming the SNP nucleotide information into numerical format and using this information as a phenotypic value for an association study. SNPs with an association of *P* ≤ 0.0001 to a chromosome were assigned to it (Additional file 1: Table S1). The physical position of each SNP marker in each wheat chromosome was determined by blasting their flanking sequences against the IWGSC Ref Seq v1.0 genome assembly [21]. Molecular data of markers associated with agronomic traits previously described [32] were also included. In the case of adaptation traits, markers were included for the genes *Rht-B1, Rht-D1* [53], *Vrn-A1* [50], *Vrn-B1, Vrn-D1* [51], *Elf3-D1* [58], *Ppd-B1* [52] and *Ppd-D1* [47]. Regarding diseases, markers for leaf rust resistance genes *Lr10* [59], *Lr34* [60] and *Lr24* [61] were incorporated. Additionally, industrial quality related markers, *Ppo-A1, Ppo-D1* [62], *R-A1, R-B1, R-D1* [63], *Glu-B1al* [64], *Wx-A1, Wx-B1* [65], *Vp1-B3* [66], *Glu-A3* [67], *PinA-D1* and *PinB-D1* [68] were included. Finally, high-molecular-weight glutenin subunit (HMW-GS) loci *Glu-A1*, *Glu-B1*and *Glu-D1* were characterized based upon the protocol described by [69].

### SNP-based haplotype construction

The SNP-based haplotype structure for each wheat chromosome was evaluated using the Haploview 4.2 software package [70]. The package defines haplotype blocks (HB) and provides the number of haplotypes and their physical length (bp) for each block, as well as the number of tagged SNPs based on the solid spine of linkage disequilibrium (LD) (Extend spine if D’ > 0.8). This means that the first and the last marker in a block are in strong LD with the intermediate markers that are not necessarily in LD with each other [18, 70]. The HBs from Haploview were converted to a table by using an in-house python script available on GitHub 2. The script transforms the table file results into a suitable input for the ’Genome Association and Prediction Integrated Tool’ (GAPIT) software [71]. Detailed information about the haplotype map constructed is given in Additional file 4: Table S3.

### Field experiments and phenotypic trait assessment

In Marcos Juárez, the field experiments were carried out during 2011 to 2016 (6 years) at INTA Experimental Station Marcos Juárez (32^∘^ 42 ^′^ S, 62^∘^ 07 ^′^ W, 114 m.a.s.l.). In Azul, the field experiments were carried out during 2013 and 2014 at the Experimental Field of the Faculty of Agronomy, Universidad Nacional del Centro de la Provincia de Buenos Aires, Azul (36^∘^ 48 ^′^ S; 59^∘^ 51 ^′^ W, 137 m a.s.l.). In Balcarce, the field experiments were carried out during crop seasons 2013, 2014 and 2015 at INTA Experimental Station Balcarce (37^∘^ 45 ^′^ S; 55^∘^ 18 ^′^ W; 130 m a.s.l.). In all experiments except Marcos Juárez 2011 and Balcarce 2013 a Randomized Complete Block Design (RCBD) with two replications was used. More detailed information of the field experiments carried out for GWAS is described in Table 6. The collection was evaluated for two crop adaptation traits: heading date (HD) and plant height (PH), and three yield component related traits: fruiting efficiency at harvest (aka spike fertility index; FEh), grain number per spike (GNS) and thousand grain weight (TGW). The HD (in days) was measured from emergence until 50% of the spike had emerged from the flag leaf [72]. The PH (in cm) was determined after maturity as the mean of 10 to 20 randomly selected plants (according to experiment) by measuring the main tiller of each plant from the ground to the top of the spike, excluding awns. At maturity, 5 to 15 random spikes (according to experiment), were sampled. They were cut at the lowest spikelet level, weighed and threshed. Spike chaff dry weight (in g) was calculated as the difference between total spike dry weight before threshing and total grain weight. The FEh (in grains g-1) was calculated at harvest as the quotient between grain number and spike chaff dry weight per sample according to [26]. The GNS was measured as the mean of the total number of grains in the selected spikes. The TGW (in g) was determined by weighing 1000 grains. In MsJz, the phenotypic data was collected using Phenobook software [73]. All the phenotypic data used for GWAS is given in Additional file 5: Table S4.

**Table 6:** Description of experiments for the Argentinean hexaploid wheat association mapping collection

Exp.	Location	Year	Blocks	Exp. unit	Traits phenotyped
1	Azul^1^	2013	2	One meter row	FEh, TGW, GNS, HD, PH
2	Azul	2014	2	One meter row	FEh, TGW, GNS, HD, PH
3	Balcarce^2^	2013^4^	1	Half meter row	FEh, TGW, GNS, HD, PH
4	Balcarce	2014	2	Plot^5^	FEh, TGW, GNS, HD, PH
5	Balcarce	2015	2	Plot	FEh, TGW, GNS, HD, PH
6	Marcos Juárez^3^	2011	1	One meter row	HD, PH
7	Marcos Juárez	2012	2	One meter row	FEh, TGW, GNS, HD, PH
8	Marcos Juárez	2013	2	One meter row	FEh, TGW, GNS, HD, PH
9	Marcos Juárez	2014	2	Plot	FEh, TGW, GNS, HD, PH
10	Marcos Juárez	2015	2	Plot	FEh, TGW, GNS, HD, PH
11	Marcos Juárez	2016	2	One meter row	FEh, TGW, GNS, HD, PH

^1^Conducted under no nutrient limitations and rainfed conditions with chemical control of pests and fungal diseases. ^2^ Conducted under no nutrient or water limitations, with chemical control of pests and fungal diseases. ^3^ Conducted under rainfed conditions without disease control. ^4^ Five sowing dates were used for all 102 cultivars; heading date information was collected for each cultivar at each sowing date and used for grouping cultivars into three groups of similar heading date. In 2014 and 2015 experiments at Balcarce, each cultivar was sown at one of each three sowing dates, in order for all cultivars to have similar heading date (around the first week of November). ^5^ Plot: 5m long seven-row plot, with a 0.2m inter-row distance

### Statistical analysis

The best linear unbiased predictors (BLUPs) of traits in each environment were obtained (except for Marcos Juárez 2011 and Balcarce 2013, where no experimental replicates were made) and were used for Pearson’s correlation analyses and broad sense heritability estimates. Broad sense heritability (*H*^2^) was estimated using the formula

1 $$ H^{2} = \sigma_{G}^{2} / \left(\sigma_{G}^{2} + \left(\sigma_{e}^{2}/r\right)\right)  $$

where $\sigma _{G}^{2}$ is the genotypic variance, $\sigma _{E}^{2}$ is the residual variance, and *r* is the number of environments. The genotype by environment variance was used as error variance $\left (\sigma _{e}^{2} = \sigma _{\mathit {GxE}}^{2}\right)$.

### Haplotype-based GWAS

GWAS were performed using 4516 haplotypes (from 1268 HB) and 495 informative SNP/gene markers anchored to the wheat genome and the SUPER method [74] implemented in the R package GAPIT [71]. In order to reduce spurious associations, genetic structure in the population level (Q values) was evaluated using R STRUCTURE software [31].The R scripts used for GWAS are available on GitHub 3 From the GWAS for each trait, we selected haplotypes/markers that were significant (*P* <0.05, marker-wise) at four environments, with at least one environment with highly significant differences (*P* <0.001). All the haplotypes/markers that satisfied these criteria are presented in Table 5. Based on the formula described in [75], the probability of a haplotype/marker being significant by chance simultaneously for four GWAS was estimated to be less than 1.25E-07(0.05x0.05x0.05x0.001). Based on this number, the probability of at least one error in the 5011 haplotypes/SNPs was estimated as 1 - (1 - 1.25E-07)^5011^ = 0.00063 (per trait).

## Supplementary information

**Additional file 1** **Table S1**. Polymorphic SNPs and gene-related molecular markers genetically and physically anchored to IWGSC Ref Seq v1.0. based on 102 Argentinean hexaploid wheats.

**Additional file 2** **Table S2**. STRUCTURE Q matrix of the 102 Argentinean hexaploid wheats cultivars.

**Additional file 3** **Figure S1**. Graphical representation of STRUCTURE Q matrix in the 102 Argentinean hexaploid wheats cultivars.

**Additional file 4** **Table S3**. Haplotype-based map used for HB characterization and GWAS in the Argentinean hexaploid wheat collection.

**Additional file 5** **Table S4**. Phenotypic data from Balcarce (2013-2015), Azul (2013-2014) and Marcos Juárez (2011-2016) for FEh, GNS, TGW, HD and PH used in GWAS.

**Additional file 6** **Table S5**. Detailed information of the significant associated Haplotypes/markes detected by GWAS for FEh, TGW, GNS, PH and HD using the 102 Argetninean hexaploid wheat collection including all the SNPs/markers ID.

## Data Availability

The datasets supporting the conclusions of this article are included within the article.

## Abbreviations

$\sigma _{E}^{2}$: Error variance
$\sigma _{E}^{2}$: Residual variance
$\sigma _{G}^{2}$: Genotypic variance
$\sigma _{GxE}^{2}$: Genotype by environment variance
bp: Base pair
BLUP: The best linear unbiased predictors
CIMMYT: Centro Internacional de Mejoramiento de Maiz y Trigo
FEh: Fruiting efficiency at harvest
GNS: Grain number per spike
GWAS: Genome wide association study
*H*^2^: Broad sense heritability
HB: Haplotype block
HD: Heading date
INTA: Instituto Nacional de Tecnologia Agropecuaria
ISBP: Insertion site-based polymorphism
IWGSC: International wheat genome sequencing consortium
LD: Linkage disequilibrium
MAF: Minor allele frequency
Mb: Mega base
PH: Plant height
r: Number of environments
RCBD: Randomized complete block design
SNP: Single nucleotide polymorphism
SSR: Simple sequence repeat
TGW: Thousand grain weight

## Footnotes

1 http://www.cerealsdb.uk.net/cerealgenomics/CerealsDB/indexNEW.php

2 https://github.com/INTABiotechMJ/haploview2gapit

3 https://github.com/INTABiotechMJ/AssociationMappingScripts
