# Supplementary material for: Haplotype block analysis of an Argentinean hexaploid wheat collection and GWAS for yield components and adaptation
Source: BMC Plant Biol. 2019 Dec 16;19:553. doi: 10.1186/s12870-019-2015-4 (PMC6916457; doi:10.1186/s12870-019-2015-4)

**Figure S1:** Structure analysis of the 102 Argentinean hexaploid wheat cultivars collection. The STRUCTURE analysis showed four hypothetical subpopulations represented by different colors.
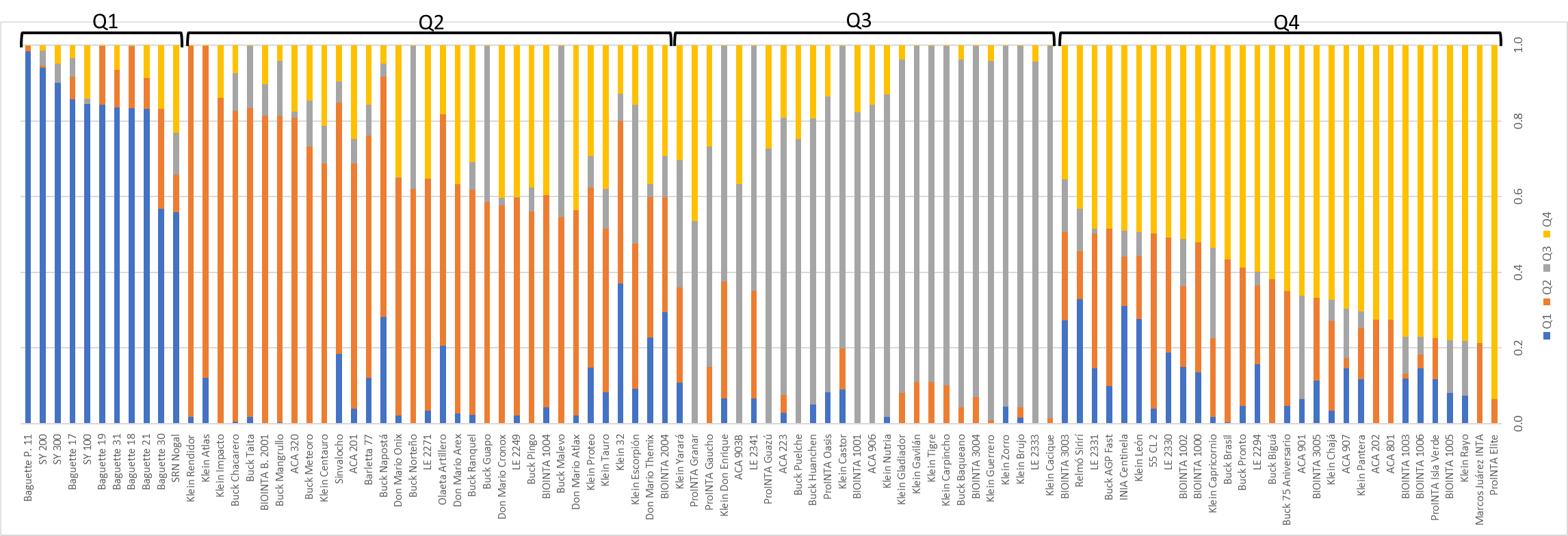

Supplement: Supplementary file 3 — Additional file 3 Figure S1. Graphical representation of STRUCTURE Q matrix in the 102 Argentinean hexaploid wheats cultivars. [file 12870_2019_2015_MOESM3_ESM.docx]
